# Visual Inspection after Acetic Acid (VIA) Is Highly Heterogeneous in Primary Cervical Screening in Amazonian Peru

**DOI:** 10.1371/journal.pone.0115355

**Published:** 2015-01-30

**Authors:** Maribel Almonte, Catterina Ferreccio, Silvana Luciani, Miguel Gonzales, Jose M. Delgado, Carlos Santos, Manuel Alvarez, Jack Cuzick, Peter Sasieni

**Affiliations:** 1 International Agency for Research on Cancer, Lyon, France; 2 Centre for Cancer Prevention, Wolfson Institute of Preventive Medicine, Queen Mary University of London, London, United Kingdom; 3 Advanced Center for Chronic Diseases, ACCDIS-FONDAP, Facultad de Medicina, Pontificia Universidad Católica de Chile, Santiago, Chile; 4 Pan American Health Organization (PAHO), Washington, DC, United States of America; 5 Dirección Regional de Salud San Martín, San Martín, Perú; 6 Instituto Nacional de Enfermedades Neoplásicas, Lima, Perú; Shanghai Jiao Tong University School of Medicine, CHINA

## Abstract

**Background:**

Conventional cytology (Pap) and visual inspection after the application of acetic acid (VIA) are currently used in primary screening in Peru. Studies suggest that the quality of VIA is highly variable. Over 36 000 women were screened with Pap and VIA in the TATI (Tamizaje y Tratamiento Inmediato de Lesiones Cervico-uterinas) project conducted in Amazonian Peru. Within a nested study to compare several screening techniques (C-TATI), a total of 5435 women were additionally screened with liquid-based cytology (LBC) and high-risk human papillomavirus testing (HR-HPV). We investigate the variation of positivity rates of VIA, Pap, LBC and HR-HPV in C-TATI and of VIA in the full TATI intervention.

**Methods:**

At the screening visit, midwives collected three cervical samples for Pap, LBC and HC2 before performing VIA. The dispersion factor “D” (D = Pearson chi-square value/degrees-of-freedom) was used to measure the variability of tests results. Within C-TATI, the variability of positivity rates of VIA, Pap, LBC and HR-HPV was also graphically assessed with box- and scatter plots by midwife and month of screening. Funnel plots and smoothed scatter plots were used to correlate the variation of VIA by the number of examinations performed by each midwife over the full TATI intervention.

**Results:**

Consistently over TATI, VIA results were highly variable, independently of the examiner, the time when the test was performed and the number of tests the examiner performed (D>6, *p*-values<0.001). In C-TATI, VIA results varied the most while those of HR-HPV varied the least (Ds>25, *p*-values<0.001 for VIA, Ds<1.6, *p*-values>0.05 for HR-HPV). No evidence for correlation between the number of VIAs done per midwife and the variability of VIA results was observed.

**Conclusion:**

The lack of over-dispersion for HR-HPV detection suggests that the variable VIA results do not reflect true variation in underlying disease, but a lack of consistency in human judgement.

## Introduction

Cervical cancer is the fourth most common female cancer in the world [1]. Cervical screening using conventional cytology (Pap) has had an impact on cervical cancer rates in many developed countries but not in the developing world [2, 3].

It has been established that infection with high-risk types of human papillomavirus (HPV) is a necessary cause for cervical cancer [4]. Both high-risk HPV detection tests (HR-HPV) and prophylactic vaccines have been developed and HPV vaccination programmes targeting young girls have been established all over the world. However, it will take several decades to see the impact of HPV vaccination, hence, cervical screening should continue. HPV testing with cytology triage may replace cytology screening soon within organised screening programmes with good-quality cytology.

The question of how best to screen populations with difficult or no access to health care remains unanswered. Visual inspection after the application of acetic acid (VIA) followed by treatment with cryotherapy has been proposed as an alternative screening approach in these settings. However, results from studies evaluating the performance of VIA have been inconsistent. In particular, several studies conducted in India and Africa have reported better VIA results than those of Latin America [5–10]. The recently published WHO guidelines for screening and treatment of precancerous lesions for cervical cancer prevention propose the use of HPV testing, if available, followed by immediate cryotherapy or by triage with VIA and then cryotherapy [11]. It is now important not only to evaluate the performance of screening tests but also to identify which tests are more prone to subjective variation (depend on providers), which suffer when used in difficult conditions in places with few specialist doctors and poor communications, and which can be used in simple algorithms to ensure that most women with lesions received adequate treatment. Over 36 000 women were screened with VIA and Pap in the “Tamizaje y Tratamiento Inmediato de Lesiones Cervico-uterinas” (TATI) project in Amazonian Peru [12]. Within a nested study that compared several screening strategies (C-TATI), 5435 women were additionally screened with liquid-based cytology and HR-HPV [7]. Here, we investigate the variation of VIA, Pap, LBC and HR-HPV results in C-TATI and of VIA in the full TATI intervention.

## Methods

The methods of TATI have been described elsewhere [7, 12, 13]. Briefly, 36 759 women aged 25–49 years residents in the San Martin region in Peru, were examined by a midwife, who took a cervical sample for a Pap and performed VIA, which was considered positive if any aceto-white lesion was observed in or close to the transformation zone and otherwise negative. Women positive on VIA were examined by a general doctor, who performed magnified VIA (VIAM), took biopsies and treated any manageable lesions with cryotherapy or referred women for colposcopy and further treatment.

TATI was conducted over 36 months. During the first three months, the logistics of the screening processes were piloted (months-3 to 0). Over the following 11 months, a comparative study (C-TATI, months 1 to 11) in which 5435 women were additionally screened with liquid-base cytology (LBC) and high-risk HPV testing (HR-HPV) done by Hybrid Capture II (hc2). Simultaneously and up until the end of TATI, women were screened with only Pap and VIA (Rest of TATI: R-TATI, months 1 to 33).

Pap and LBC reported as ASCUS or worse (Pap ASCUS, LBC ASCUS) were considered positive and the standard threshold of 1 (RLU/co) of hc2 was used to identify HR-HPV positive samples. No HPV genotyping was done in this study. Cervical intraepithelial neoplasia grade 2 or worse (CIN2+) was the main disease outcome of the study.

### VIA training

Initial training was offered to 19 midwives and 12 general doctors on VIA [7, 13]. The training included theoretical and practical sessions on performing VIA and collecting cervical samples for Pap, general aspects of female anatomy, cervical cancer prevention, diagnosis and management of sexually transmitted infections and communication and counselling skills. Photographs of cervices were reviewed throughout the course. Trainees practiced VIA first on anatomical models and then examined women under direct supervision of trainers. General doctors were additionally trained on VIAM and treatment with cryotherapy. A total of 42 midwives and 25 doctors were trained in TATI over three training courses of one week each.

### Statistical analysis

We used the dispersion factor (D) to measure the variability of the screening tests results by a number of factors: age (in five-year bands), midwife who performed VIA and month of screening, overall and separately by sub-study (C-TATI vs. R-TATI). D was defined as the ratio of the Pearson chi-square value of a 2 x m table (2 = positive or negative, m = number of levels of factor of interest) divided by the degrees of freedom (d.f.= n = m-1). Since the expected value of a random variable *X* with a chi-square distribution with “n” d.f. is “n”, the expected value of D under the null hypothesis of equal underlying properties in each strata is approximately 1, i.e. E(D) = E(*X*)/n = 1. Thus, D values close to zero will represent minimal dispersion (less than random variation) and highest values of D, further above unity, will represent over dispersion.

Positivity rates (PR) of VIA, Pap ASCUS, LBC ASCUS and HR-HPV were calculated overall, by age, by month of screening and by midwife who collected the samples and performed VIA. The PR of each midwife (42 different VIA PRs) participating in TATI was calculated including the results of all examinations performed throughout the intervention and grouped into Group 1: VIA performed by 19 midwives who participated in C-TATI (19 VIA PRs) and Group 2: VIA performed by 23 midwives who participated in R-TATI but not in C-TATI (23 VIA PRs). As the 19 C-TATI midwives in Group 1 screened women in C-TATI and in R-TATI in parallel (in alternative scheduled clinics), their PRs were also calculated separately for examinations done in C-TATI (19 PRs) and R-TATI (19 PRs). Next, the overall PR within each group was calculated summing up the results of all VIAs performed in the group with corresponding binomial 95% confidence intervals (95%CI). A test for trends was used to compare VIA PRs over time (year 1 = months 1–11, year 2: 12–23, and year 3 = 24–33) in R-TATI.

We graphically assessed the variation of PRs with: 1) box plots to compare the variation of PRs of screening tests in C-TATI, 2) error-bar plots to summarise the difference between VIA PRs performed by the same midwife during the first year of intervention in C-TATI and R-TATI, 3) funnel plots to evaluate the extent to which the variability of VIA results could be explained by the number of examinations performed by each midwife and 4) smoothed scatter plots to evaluate whether the variability of VIA results could be explained by a learning curve and if so to identify a minimal number of VIA examinations required to obtain less variable results [14].

Both funnel plots and smoothed scatter plots allow visualisation of the variation of VIA PRs taking into account the number of VIAs performed. Funnel plots were graphed in two steps: 1) scatter plots of VIA PRs by the number of VIAs performed, and 2) funnels with 95%CIs (control limits) were constructed around the overall VIA PR. In these graphs, the areas within the control limits represent the amount of variation expected. Thus, the higher the number of PRs outside the control limits, the more over-dispersed the data are; and the most closely clustered the PRs are to the overall rate, the less random variation. A running line was used to smooth the scatter plots for each midwife and obtain the smoothed scatter plots.

All statistical analyses were done using the Stata software, version 12.

### Ethical Considerations

The TATI project was approved by the Ethical Committee of the Ministry of Health of Peru and the Ethics Committee of the Pan American Health Organization. Written informed consent was obtained from all participants by trained midwives.

## Results

**Figure 1:**
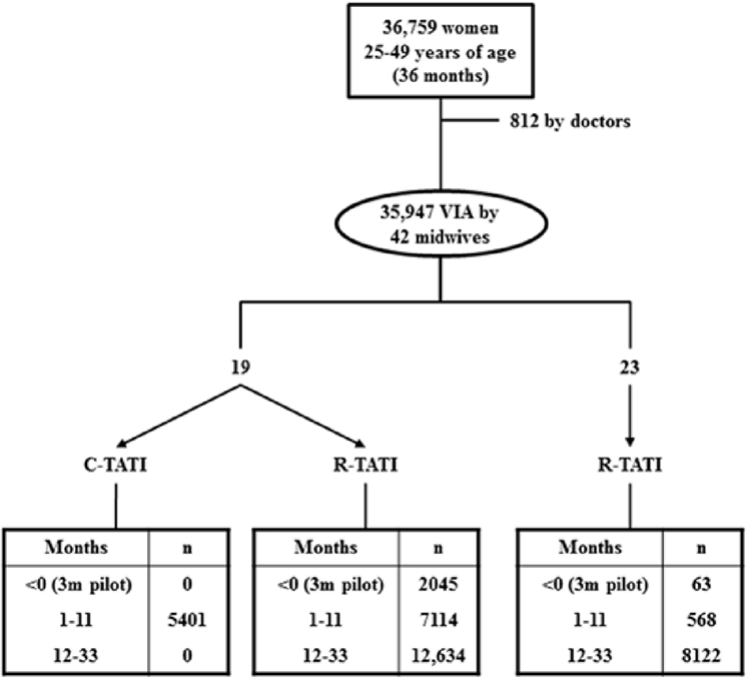
Diagram of the TATI project with the number of visual examinations performed by midwives in the pilot phase (months-3–0), during the time of the comparative study (months 1–11) and after the comparative study (months 12–33). VIA = Visual inspection after the application of acetic acid. C-TATI = Comparative screening study (months 1–11). R-TATI = rest of the TATI intervention (months 1–33).

After excluding 812 VIAs performed by doctors, a total of 35 947 VIAs were performed by 42 midwives over 36 months. During the pilot phase of the intervention, 2108 women were screened with Pap and VIA. Over the next 11 months, 19 midwives participating in C-TATI performed 12 515 VIA examinations, 5401 within C-TATI and 7114 within R-TATI; while 568 VIAs were performed by nine of the 23 midwives who only participated in R-TATI. Once C-TATI finalised, VIA was performed in 20 756 women (61% of these examinations were done by the 19 C-TATI midwives). From here on, results are based on 33 839 women who were screened by a midwife after the pilot phase (Fig. 1).

**Figure 2:**
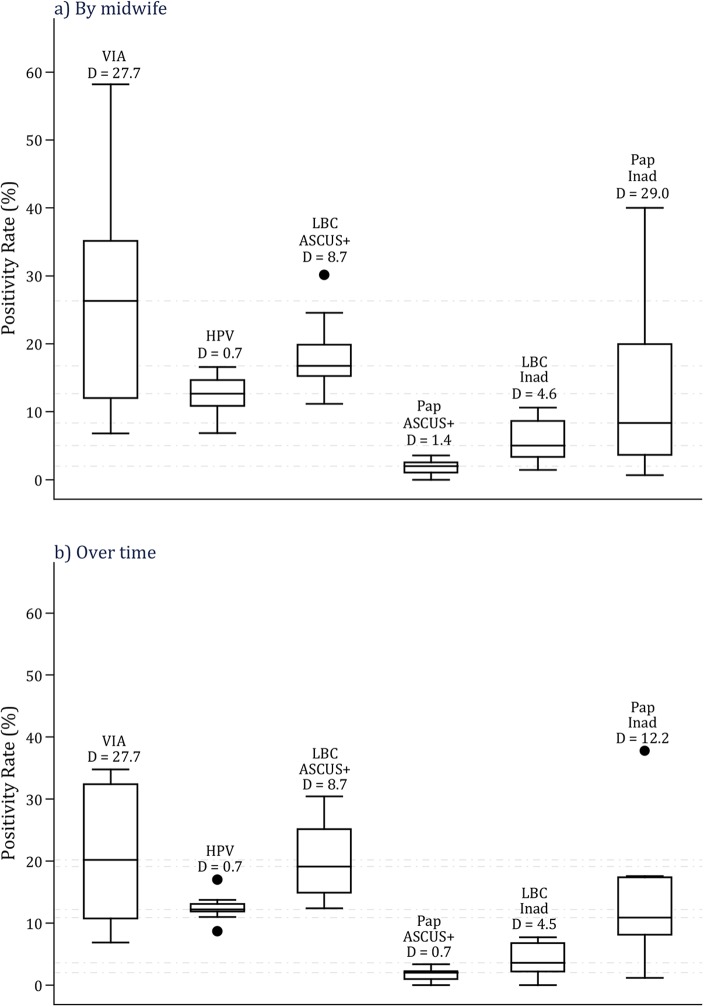
Box plots of positivity rates of VIA, HPV, LBC and Pap and of inadequate rates of LBC and Pap in C-TATI. VIA = Visual inspection after the application of acetic acid. HPV = Human papillomavirus. LBC = Liquid-based cytology. C-TATI = Comparative screening study (months 1–11). D = Dispersion factor. ASCUS+ = Atypical squamous changes of undetermined significance or worse cytological abnormalities. Inad = Inadequate.

In C-TATI (n = 5401), the overall PRs were 24.2% for VIA, 2.1% for Pap ASCUS+, 17.9% for LBC ASCUS+ and 12.6% for HR-HPV testing. Each of these tests were positive in 36 (43%), 32 (39%), 64 (77%) and 79 (95%) of the 83 CIN2+ detected cases. The PRs of VIA and HR-HPV testing decreased with increasing age while those for Pap and LBC increased with increasing age, mostly due to an increasing trend of moderate dysplasia and worse abnormalities by age (all *p*-values for trends were highly significant, S1 Table). Fig. 2 shows box plots of the variation of PRs of screening tests by midwife and over time. VIA PRs varied the most (D = 27.8 and 27.7 by midwife and over time, *p*-values<0.001). In contrast, HR-HPV testing was the most homogeneous test (D = 1.5 and 0.7,*p*-values>0.05, respectively). PRs of LBC ASCUS+ also varied significantly (D = 3.1 and 8.7, *p*-values<0.001) while those of Pap ASCUS+ varied little (D = 1.4 and 0.7, *p*-values>0.05). After taking into account the low rate of disease detected by Pap (S1 Table), we repeated the analysis on inadequate rates (IR) of both LBC and Pap. This time the Pap IRs were largely more heterogeneous (D = 29.0 by midwife and 12.2 over time, *p*-values<0.001) than LBC IRs and PRs of LBC and HR-HPV (data not shown). We also repeated the analysis on positivity rates of VIA and Pap in R-TATI. The results were similar, VIA PRs were more heterogeneous than Pap smear PRs, however, the results of both tests were highly variable (data not shown).

A summary of VIA PRs per midwife by group of midwives is presented in Table 1. Overall, midwives performed between 30 and 2346 VIAs. Two midwives performed less than 100 VIA exams (one did 30, the other 80), 43 performed between 113 and 1962 VIAs each and the remaining three over 2000 VIAs (2174, 2256 and 2346, respectively). The overall VIA PR per midwife in Group 1 was 16% (95%CI: 15.5%, 16.5%) and was significantly lower than that of VIA performed by midwives in Group 2 (23.5%, 95%CI: 22.6%, 24.4%). In contrast, the dispersion factor of VIA results of midwives in Group 1 was almost double that of midwives in Group 2 (D = 48.2 and D = 27.0, *p*-values<0.001), although both dispersion factors were highly significant. The results did not change after excluding midwives who performed less than 100 or more than 2000 VIA examinations. Surprisingly, the PR of midwives in Group 1 was significantly higher when they screened women in C-TATI (24.2%, 95%CI: 23.1%, 25.4%) than when they screened women in R-TATI (13.7%, 95%CI: 13.3%, 14.2%), and higher than the overall PR of all midwives together (17.9, 95%CI: 17.5%, 18.3%). In view of these results, we compared PRs in C-TATI and R-TATI of the 19 midwives in Group 1 by plotting the difference between the two positivity rates (C-TATI—R-TATI) with 95%CIs. The difference was negative (decrease of 0.4%) only for one midwife while the rest had higher PRs in C-TATI than in R-TATI (mean difference: 13.6%, 95%CI: -10.9%, 38.2%). Thus, midwives assigned more frequently a positive result within C-TATI than within R-TATI (S1 Fig.).

**Table 1:** Variation of VIA positivity rates per midwife over the 33 months of the TATI intervention by group of midwives.

**Group of midwifes (No.)**	**No. women screened**	**No. VIA/midwife**		**%VIA positive/midwife**		**Dispersion factor^1^**
		**Mean**	**Range**	**Overall (95%CI)**	**Range**	
Group 1 (19)						
C-TATI	5401	284.3	122–544	24.2 (23.1–25.4)	6.8–58.2	27.8
R-TATI	19,748	1039.4	357–2063	13.7 (13.3–14.2)	7.0–24.4	30.9
All group 1	25,149	1323.6	638–2346	16.0 (15.5–16.5)	7.2–27.2	48.2
Group 2 (23)						
R-TATI	8690	377.8	30–983	23.5 (22.6–24.4)	7.1–43.4	27.0
All midwives (42)	33,839	554.7	30–2346	17.9 (17.5–18.3)	7.1–43.4	43.0

C-TATI = Comparative screening study (months 1–11). R-TATI = rest of the TATI intervention (months 1–33). Group 1: 19 midwives who participated in C-TATI and R-TATI. Group 2: 23 midwives who did not participate in C-TATI. 95%CI = 95% confidence interval.

^1^All dispersion factors were highly significant (*p*-value<0.001)

Table 2 shows the VIA PRs of midwives by group and year of the intervention, when they screened women only in R-TATI. VIA PRs of midwives in Group 1 were consistently lower than those of midwives in Group 2 (at least 7% difference between overall rates, statistically significant each year). There was a consistent increasing trend of the overall PRs per group of midwives and in both groups combined from the first year to the second year, and a consistent small decrease from the second to the third year (*p*-for-trend<0.001). However, all Ds were once again highly significant. It is worth noting that five midwives in Group 2 performed less than 50 VIAs in the first year, hence the range of VIA PRs varied between 0% and 67% (D = 8.6). After excluding extreme values, the results did not change (range: 8.6% to 39.5%, D = 12.5, *p*-value<0.001).

**Table 2:** Variation of VIA positivity rates per midwife by group of midwives and year of intervention within R-TATI.

**Group of Midwives**		**Year 1 February-December**			**Year 2 January-December**			**Year 3 January-September**		
	**No. VIA^a^ (No. Mid.)**	**Mean No. VIA**	**% VIA positive/midwife Overall (95%CI)**	**D^b^**	**No. VIA^a^ (No. Mid.)**	**% VIA positive/midwife Overall (95%CI)**	**D^b^**	**No. VIA^**a**^ (No. Mid.)**	**% VIA positive/midwife Overall (95%CI)**	**D^b^**
Group 1 R-TATI	7114 (19)	7114 (19)	12.2 (11.5–12.0)	7.4	6933 (19)	15.0 (14.2–15.8)	12.5	5701 (18)	14.1 (13.2–15.0)	17.5
Group 2 R-TATI	568 (9)	568 (9)	21.1 (17.8–24.7)	8.6	3032 (19)	27.1 (25.6–28.8)	13.7	5090 (21)	21.5 (20.4–22.7)	20.7
All	7682 (28)	7682 (28)	12.9 (2.2–13.7)	9.9	9965 (38)	18.7 (17.9–19.5)	19.3	10,791 (39)	17.6 (16.9–18.3)	21.9

R-TATI = rest of the TATI intervention (months 1–33). Group 1: 19 midwives who participated in C-TATI and R-TATI. Group 2: 23 midwives who did not participate in C-TATI. Mid. = Midwives. D = Dispersion factor. 95%CI = 95% confidence interval.

^**a**^ Overall number of VIA examinations performed by midwives in that group during that year

^**b**^ All dispersion factors were highly statistically significant (*p*-value < 0.001)

**Figure 3:**
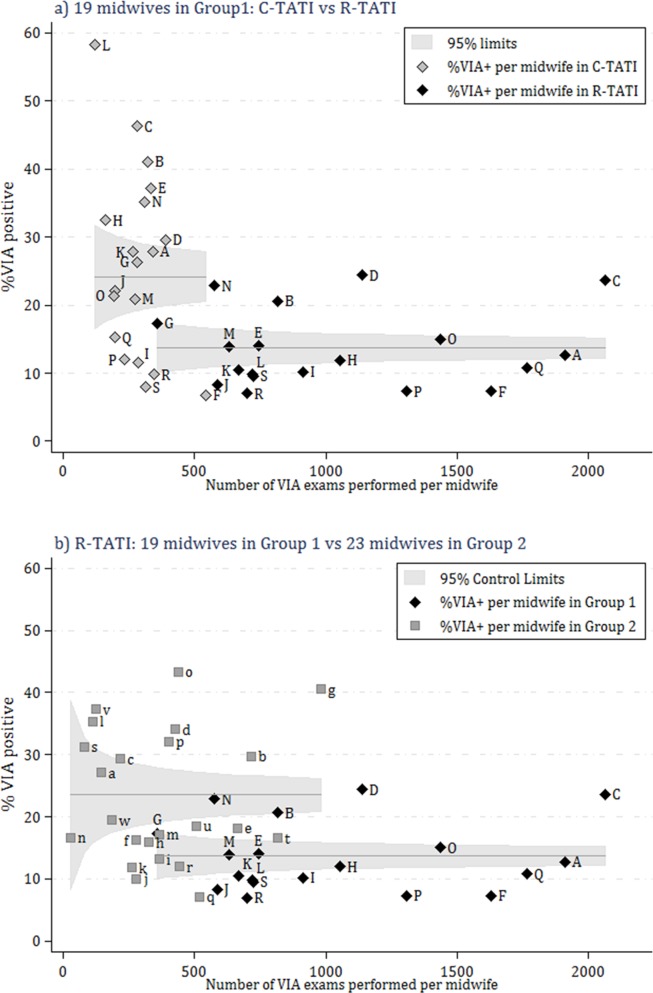
Funnel plots of the variation of positivity rates of VIA performed by midwives over 33 months of the TATI intervention. C- TATI = Comparative screening study (months 1–11). R-TATI = rest of the TATI intervention (months 1–33). Group 1: 19 midwives who participated in C-TATI and R-TATI. Group 2: 23 midwives who did not participate in C-TATI. Positivity rates of VIA performed by midwives in Group 1 are represented by capital letters “A” to “S”. Positivity rates of VIA performed by midwives in Group 2 are represented by small letters “a” to “w”.

In summary, independently of whether: i) VIA was performed in C-TATI or in R-TATI, ii) VIA was performed by midwives in Group 1 or Group 2, or iii) VIA was performed in the first, second or third year; VIA positivity rates were significantly and consistently heterogeneous (D>7, *p*-values<0.001 for all possible combinations). Fig. 3a shows the variation of VIA PRs of each of the 19 Group 1 midwives (denoted by capital letters “A” to “S”) when they screened women in C-TATI (left, in grey diamonds) and in R-TATI (right, in black diamonds). Within C-TATI, five VIA PRs were above 35% (B, C, E, L and N) and three below 10% (F, R and S) while in R-TATI all rates were below 25% with six of them below 10% (F, J, L, P, R and S). Despite the apparent correlation of VIA positivity with the number of exams done (as midwives performed more VIAs, their PRs became more tightly clustered around the overall VIA PR); the majority of VIA PRs lied outside the 95% control limits and only six (A, G, J, K, M and O) in C-TATI and five (A, E, H, M and O) in R-TATI were within the expected range. There was a substantial 43% reduction in the overall VIA PR within R-TATI in comparison to C-TATI, therefore; we decided to repeat this analysis, including all midwives performing VIA only in R-TATI (Fig. 3b). This time, Group 2 VIA PRs (denoted by small letters “a” to “w”) were plotted in grey squares and those of Group 1 (denoted by letters “A” to “S”) in black diamonds as in Fig. 3a. The pattern of VIA PRs performed in R-TATI by midwives in Group 2 who did not participate in C-TATI, was somehow similar to that of VIA PRs in C-TATI shown in Fig. 3a. This time, the rates of 13 of the 23 midwives in Group 2 were lower than 20%; however, only five positivity rates were within the expected range (a, c, n, s and w).

**Figure 4:**
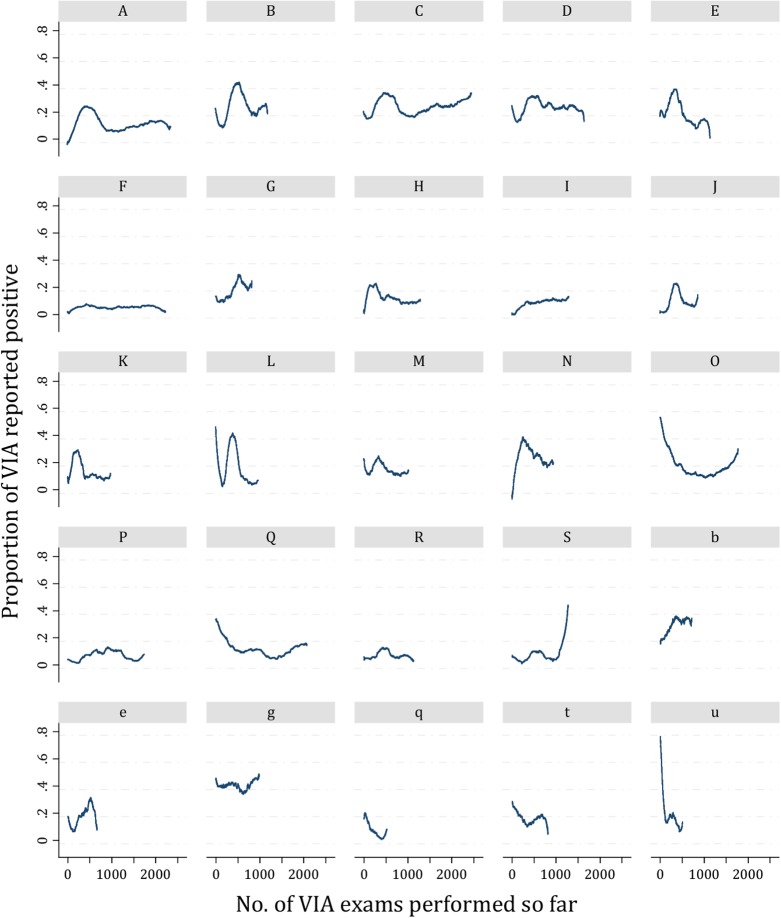
Proportion of VIA exams reported positive according to the number of VIA exams carried out by midwives who performed 500 or more VIA exams throughout the intervention. Proportion of VIA exams reported positive by midwives in Group 1 are represented by capital letters and those of midwives in Group 2 by small letters.

Fig. 4 shows the proportion of VIAs reported positive over time (i.e. according to the number of VIAs carried out) for each of the 25 midwives who performed at least 500 VIAs. For instance midwife A was initially calling very few VIAs positive. By the time she has performed 500 VIAs she was calling out 20% positive. The proportion positive fell sharply to about 8% after 1000 examinations and then rose slowly to about 19% with 2000 examinations. Beyond 2000 the proportion fell back slightly to about 15%. The different patterns of the smoothed curves confirmed the variation of VIA results and suggested that this variability could not be explained by the number of VIAs performed. No evidence for a learning curve or a minimal number of VIAs needed to achieve proficiency was observed.

## Discussion

We have clearly demonstrated that most of the variation in VIA PRs in TATI may be attributed to lack of consistency in interpretation. There was substantial variability in the proportion of examinations called positive by month and by midwife who performed the test in C-TATI; in contrast HR-HPV testing results were consistently homogeneous. These results suggested that the variation in VIA PRs was not due to disease variation (1.6% overall rate of CIN2+, range: 1.0–4.4, D = 0.20) but to the subjective nature of the test.

Surprisingly, despite the fact that the 19 C-TATI midwives screened women in C-TATI and in R-TATI in parallel, the overall PR of VIA performed by them in C-TATI (24.2%) was twice the PR when they screened women in R-TATI (13.7%). Indeed, 18 of 19 VIA PRs in C-TATI were higher than in R-TATI during the first 11 months of the intervention. One potential explanation for these differences could be bleeding of the cervix associated to additional sampling (LBC and HPV after Pap) in C-TATI. The bleeding and the high prevalence of vaginal infections may have interfered with the visualisation of the transformation zone, affecting VIA performance, despite this, VIA results in C-TATI were similar to those of other studies [7, 15–19].

The minimal number of VIAs and the average monthly rate of VIAs that a provider needs to achieve VIA proficiency have not yet been determined [2]. On first inspection, our analysis suggested that as the number of VIAs performed increases, the examiner gets more expertise on the test, is able to better distinguish aceto-white lesions with higher likelihood of being true lesions, and grants less false positive results (Fig. 3). This was reinforced by similar patterns of variation among VIA results of the 19 C-TATI midwives in C-TATI (Fig. 3a) and those of the 23 R-TATI midwives in R-TATI (Fig. 3b). However, when we plotted the proportion of VIA positive results of midwives who at least performed 500 exams, there were no consistent patterns of VIA PRs, contradicting our previous observations and that of a learning curve of VIA after initial training [19].

Similarly to previous studies, the high variability of VIA results per midwife was not explained by lack of adequate and uniform training [18, 20–23]. In our study, midwives were intensively trained over a week in which they performed VIA under rigorous supervision. Furthermore, whenever women had high-grade cytology and negative VIA, they were re-examined by the midwife and an expert gynaecologist together and reasons for not visualising lesions were discussed.

Our study has some limitations. First, we were not able to contrast positivity rates with disease ascertainment in R-TATI because only Pap and VIA were used to refer women and undoubtedly several cases of CIN2+ were missed. However, we performed this comparison within C-TATI and we showed that the high variation of VIA results was not due to disease variation but to human judgement. Second, midwives started performing VIA at different time points in TATI and only four midwives performed VIA each month of TATI, possibly affecting the likelihood of achieving proficiency. However, we evaluated the variation of VIA results performed in a month per midwife among midwives who at least performed 500 examinations, and found no consistent pattern among the proportion of VIA positive results of midwives. Clearly, the strongest evidence would have come from having multiple midwives examining each woman blinded to each other’s opinion or by assigning women to midwives at random. Neither of such options was practical, and so we have relied on analysis of data that could be subject to confounding. Nevertheless, our study is the first to compare the VIA results of 42 midwives who altogether performed more than 36 000 VIAs within their routine clinical activities in a low-resource setting, and the results are robust to the method of analysis and are likely to reflect true variation.

Despite inconsistent results on its performance, VIA is usually the main screening technique in use in low-resource settings, partly because it is the only simple low-cost test available and partly because VIA results are known immediately allowing see-and-treat schemes. New HR-HPV low-cost tests may soon become available and HPV-and-treat or HPV-VIA-and-treat schemes are already being considered as potential alternatives to VIA-and-cryotherapy. The results presented here will contribute to the evidence needed by decision makers when choosing cervical screening algorithms appropriate for their local needs.

## Supporting Information

S1 Fig Difference in positivity rates of VIA in C-TATI and R-TATI performed by midwives who participated in C-TATI. C- TATI = Comparative screening study (months 1–11). R-TATI = rest of the TATI intervention (months 1–33). Difference between positivity rates of VIA (C-TATI—R-TATI) of each midwife is represented by a capital letter. The vertical dotted line represents the mean difference (13.6%, 95%CI: -10.9%, 38.2%).
(TIF)

S1 Table Positivity rates of screening tests and number of CIN2+ detected in the comparative study C-TATI. C-TATI = Comparative screening study (months 1–11). CIN2+ = Cervical intraepithelial neoplasia grade 2 or worse. VIA = Visual inspection after the application of acetic acid. Pap ASCUS = Pap result ASCUS or worse. LBC ASCUS = Liquid-based cytology result ASCUS or worse. HR-HPV = High-risk human papillomavirus testing.
(DOCX)
