# Supplementary figures and images for: Visual Inspection after Acetic Acid (VIA) Is Highly Heterogeneous in Primary Cervical Screening in Amazonian Peru
Source: PLoS One. 2015 Jan 30;10(1):e0115355. doi: 10.1371/journal.pone.0115355 (PMC4312028; doi:10.1371/journal.pone.0115355)

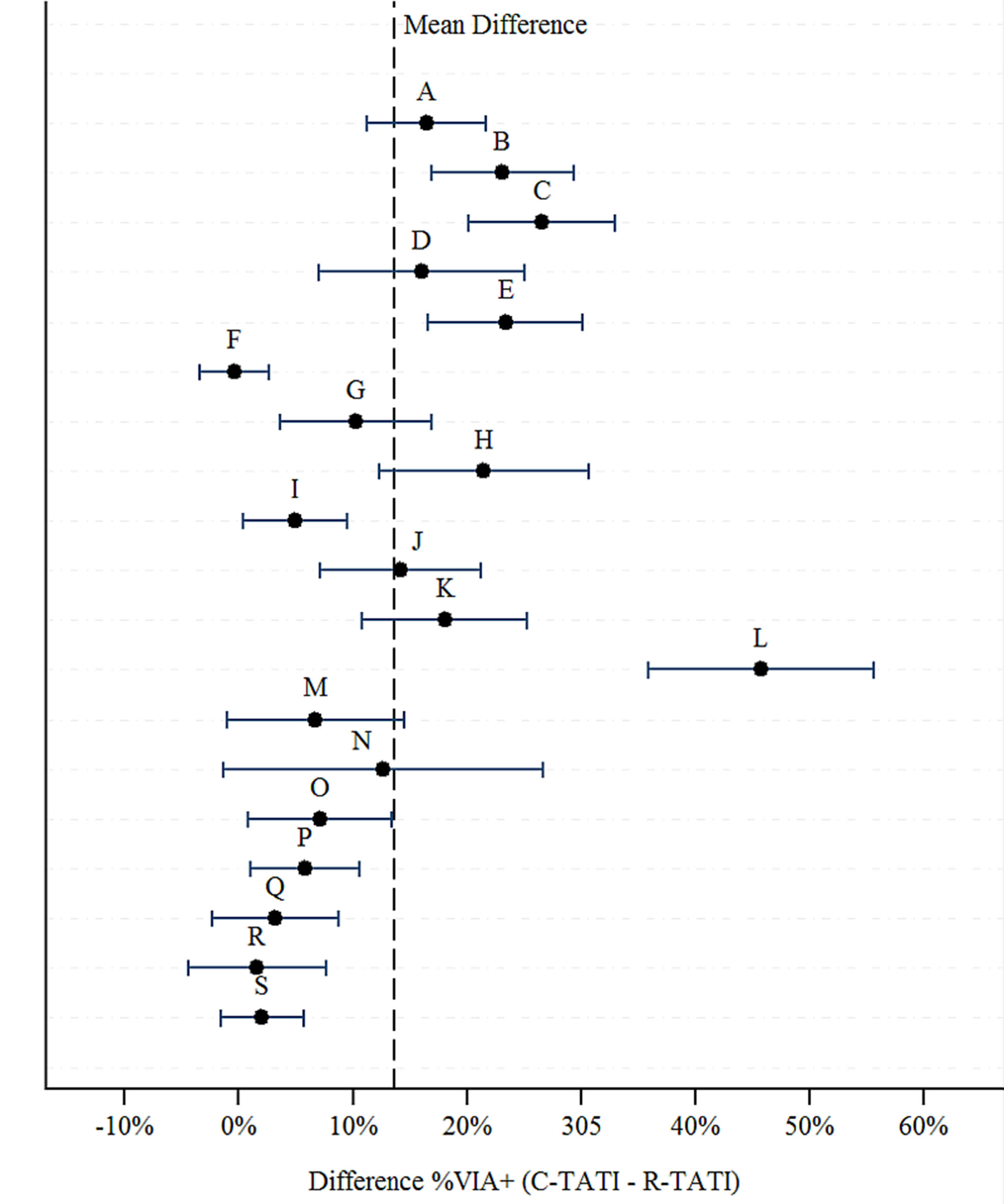

Supplement: S1 Fig — C- TATI = Comparative screening study (months 1–11). R-TATI = rest of the TATI intervention (months 1–33). Difference between positivity rates of VIA (C-TATI—R-TATI) of each midwife is represented by a capital letter. The vertical dotted line represents the mean difference (13.6%, 95%CI: -10.9%, 38.2%). (TIF) [file pone.0115355.s001.tif]
