# Supplementary material for: Visual Inspection after Acetic Acid (VIA) Is Highly Heterogeneous in Primary Cervical Screening in Amazonian Peru
Source: PLoS One. 2015 Jan 30;10(1):e0115355. doi: 10.1371/journal.pone.0115355 (PMC4312028; doi:10.1371/journal.pone.0115355)
Supplement: S1 Table — C-TATI = Comparative screening study (months 1–11). CIN2+ = Cervical intraepithelial neoplasia grade 2 or worse. VIA = Visual inspection after the application of acetic acid. Pap ASCUS = Pap result ASCUS or worse. LBC ASCUS = Liquid-based cytology result ASCUS or worse. HR-HPV = High-risk human papillomavirus testing. (DOCX) [file pone.0115355.s002.docx]

Table S1. Positivity rates of screening tests and number of CIN2+ detected in the comparative study C-TATI

|  |  | No. | Positivity rates (%) | | | |
| --- | --- | --- | --- | --- | --- | --- |
|  |  | screened | VIA | Pap ASCUS | LBC ASCUS | HR-HPV |
| Age |  | (n = 5401*) | (n = 1309) | (n = 96) | (n = 911) | (n = 682) |
| 25-29 | | 1461 | 24.8 | 1.3 | 15.8 | 15.5 |
| 30-34 | | 1461 | 25.7 | 2.0 | 16.7 | 12.5 |
| 35-39 | | 1213 | 25.4 | 2.0 | 19.8 | 11.8 |
| 40-44 | | 768 | 21.6 | 3.1 | 18.5 | 11.5 |
| 45-49 | | 498 | 19.7 | 3.1 | 21.5 | 8.6 |
| **Overall positivity rates** | | | **24.2** | **2.1** | **17.9** | **12.6** |
| CIN2+ | No. | 83 | 36 | 32 | 64 | 79 |
|  | % | 100 | 43.4 | 38.6 | 77.1 | 95.2 |

* Excluding 34 women in whom VIA was performed by medical doctors.
